# Melioidosis in South Asia (India, Nepal, Pakistan, Bhutan and Afghanistan)

**DOI:** 10.3390/tropicalmed3020051

**Published:** 2018-05-22

**Authors:** Chiranjay Mukhopadhyay, Tushar Shaw, George M. Varghese, David A. B. Dance

**Affiliations:** 1 Department of Microbiology, Kasturba Medical College, Manipal Academy of Higher Education, Manipal 576104, India; tusharshaw1990@gmail.com; 2 Center for Emerging and Tropical Diseases, Manipal Academy of Higher Education, Manipal 576104, India; 3 Department of Infectious Diseases, Christian Medical College, Vellore 632004, India; georgemvarghese@hotmail.com; 4 Lao-Oxford-Mahosot Hospital-Wellcome Trust Research Unit, Vientiane, Laos; David.d@tropmedres.ac; 5 Centre for Tropical Medicine and Global Health, University of Oxford, Oxford OX1 2JD, UK; 6 Faculty of Infectious and Tropical Diseases, London School of Hygiene and Tropical Medicine, London WC1E 7HT, UK

**Keywords:** *Burkholderia pseudomallei*, India, melioidosis, South Asia

## Abstract

Despite the fact that South Asia is predicted to have the highest number of cases worldwide, melioidosis is a little-known entity in South Asian countries. It has never been heard of by the majority of doctors and has as yet failed to gain the attention of national Ministries of Health and country offices of the World Health Organization (WHO). Although a few centers are diagnosing increasing numbers of cases, and the mortality documented from these institutions is relatively high (nearly 20%), the true burden of the disease remains unknown. In India, most cases have been reported from southwestern coastal Karnataka and northeastern Tamil Nadu, although this probably simply reflects the presence of centers of excellence and researchers with an interest in the disease. As elsewhere, the majority of cases have type 2 diabetes mellitus and occupational exposure to the environment. Most present with community-acquired pneumonia and/or bacteremia, especially during heavy rainfall. The high seropositivity rate (29%) in Karnataka and isolation of *B. pseudomallei* from the environment in Tamil Nadu and Kerala confirm India as melioidosis-endemic, although the full extent of the distribution of the organism across the country is unknown. There are limited molecular epidemiological data, but, thus far, the majority of Indian isolates have appeared distinct from those from South East Asia and Australia. Among other South Asian countries, Sri Lanka and Bangladesh are known to be melioidosis-endemic, but there are no cases that have conclusively proved to have been acquired in Nepal, Bhutan, Afghanistan or Pakistan. There are no surveillance systems in place for melioidosis in South Asian countries. However, over the past two years, researchers at the Center for Emerging and Tropical Diseases of Kasturba Medical College, University of Manipal, have established the Indian Melioidosis Research Forum (IMRF), held the first South Asian Melioidosis Congress, and have been working to connect researchers, microbiologists and physicians in India and elsewhere in South Asia to raise awareness through training initiatives, the media, workshops, and conferences, with the hope that more patients with melioidosis will be diagnosed and treated appropriately. However, much more work needs to be done before we will know the true burden and distribution of melioidosis across South Asia.

## 1. Introduction

Melioidosis is a potentially fatal illness which is caused by the soil saprophyte and biothreat agent *Burkholderia pseudomallei*. It is known to be highly endemic in northeast Thailand and northern Australia, where annual incidence rates ranging from 4 to 41.7 cases per 100,000 population [1] have been reported. A recent modeling study predicted that as many as 165,000 people may be infected each year worldwide, with South Asia having the highest burden of the disease (44% of all cases) [2]. India is the largest country in South Asia and, having a suitable environment and an enormous diabetic population, might well be a ‘hotspot’ for the disease. However, the true melioidosis burden in India is unknown due to limited awareness and laboratory constraints throughout the country. Clinical diagnosis is exceptionally challenging due to the varied clinical presentations, especially as the disease can mimic other infections such as tuberculosis [3], which is highly endemic in resource-poor countries such as India.

The majority of the Indian population resides in rural settings and might readily acquire the infection through direct contact with soil and water. However, they have limited access to hospitals where there are well-equipped microbiology laboratories and well-trained professionals to diagnose the disease. The present-day situation in India is similar to that in Thailand in the mid-1980s when melioidosis started to be diagnosed increasingly as microbiology services improved. In countries such as India, limited awareness among clinicians and microbiologists about the disease has led to misdiagnosis and inappropriate treatment. Furthermore, the spectrum of disease manifestations ranges from the acute septicemic form to a chronic granulomatous form, and hence it mimics other endemic infections with which it may be confused. Although there has been a plethora of melioidosis case reports and short series published from India, there are no voluntary or mandatory surveillance systems to notify the cases, and so there has been limited evidence to encourage governments to formulate melioidosis-specific public health policies.

The challenges to the laboratory in diagnosing melioidosis are manifold. Wrinkled *B. pseudomallei* colonies are frequently thought to be aerobic spore bearing bacteria, which are relatively common environmental contaminants, and discarded. Any oxidase-positive Gram-negative, non-lactose fermenting bacillus is likely to be considered as a ‘pseudomonad’ or ‘*Pseudomonas* sp.’, which is unlikely to be identified further to the species level. Even commercial identification kits and modern automated systems (e.g., API 20NE, VITEK 2, and MALDI-TOF) are not 100% reliable for identifying *B. pseudomallei*, especially in inexperienced hands [4]. However, awareness of the disease has recently been increasing in this subcontinent and it is being diagnosed more frequently, especially in India, Sri Lanka and Bangladesh. The current review aims to bring into focus a much neglected ‘killer’ disease from this region, focusing on India, Pakistan, Bhutan, Nepal, and Afghanistan, as Bangladesh and Sri Lanka are covered in separate reviews in this Special Issue.

## 2. History

Following the initial recognition of melioidosis by the British pathologist Alfred Whitmore and his Indian assistant, CS Krishnaswami, in Myanmar in 1911, the first case in South Asia was confirmed in Ceylon, now Sri Lanka, in 1927. The first case from India was not diagnosed until 1953, in a 40-year-old Scottish mining engineer suffering from prolonged fever, multiple abscesses and septicemia, who became infected while working in the central part of the country but was diagnosed post-mortem following his repatriation to Scotland [5]. Another fatal exported case was diagnosed in Switzerland in 1988 in a 40-year-old woman who had traveled extensively in India and Nepal [6]. It thus appeared likely that melioidosis was endemic within India.

The first indigenous case from India was detected in Mumbai in 1991 [7], which was followed by a long period of silence. Meanwhile, the epidemics thought to be the bubonic plague in Beed (Maharashtra) and pneumonic plague in Surat (Gujarat), which occurred in the mid-1990s, led to the suggestion that at least some of the cases may have been melioidosis [8], although this was questioned [9] and was never confirmed officially. This was followed by a series of publications from the Christian Medical College in Vellore, near the south-eastern coast of India, which raised the likelihood of melioidosis being endemic in the country and the probability of it being a silent killer due to incorrect diagnosis and treatment [10,11,12,13,14]. A small serosurvey carried out around the same time suggested that a proportion of the local rural population had evidence of exposure to *B. pseudomallei* [15].

## 3. Review of Melioidosis Cases and Presence of *B. pseudomallei* (Animal/Human/Environment)

We performed a PubMed search using the keywords ‘*melioidosis*’, ‘*pseudomallei*’, and ‘*India*’, and reviewed the available references published between January 1991 and March 2018. Initially, titles and abstracts were screened and articles identified as possibly relevant were reviewed as full text. The reference lists of included articles were assessed for further relevant publications. A similar search strategy was applied for Nepal, Pakistan, Afghanistan, and Bhutan. In addition, cases reported from India were also identified from the website http://www.melioidosis.info. Duplicate cases were screened and removed manually. Additional cases were identified from the personal EndNote database of one of the authors (DABD), as many reports published in non-indexed journals were not available on PubMed. Further, more detailed information was obtained from our own laboratory database regarding cases diagnosed in Manipal since 2002, and Vellore since 2008.

### 3.1. India

**Figure 1:**
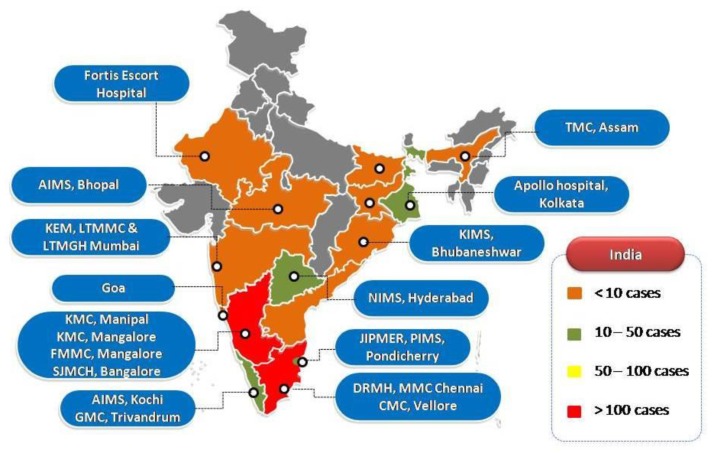
Cases reported from different parts of India and centers equipped to diagnose cases of melioidosis.

The literature search revealed 190 articles (File S1) comprising case reports or series relating to 583 individual patients with melioidosis from different parts of India (Table 1 and Figure 1). The greatest number of cases were reported from Karnataka (306), followed by Tamil Nadu (146). Figure 1 shows clearly that these cases were predominantly diagnosed in coastal areas, probably reflecting the fact that the majority of the best-resourced hospitals are located in these regions. The ages ranged from 0 to 84 years, although many publications did not provide enough detail to calculate overall medians and interquartile ranges. A substantial majority of the patients were male, which may be attributable to men being more likely to be involved in occupations involving soil contact, but also to inequality in access to the health care between men and women. Diabetes was the most common predisposing factor reported (391/559, 70%). Ceftazidime was the most common antibiotic used in the intensive phase and cotrimoxazole for eradication therapy. The overall reported mortality was 95 (17%), although this may have been an underestimate as there were many cases in which the outcome was not reported (Table 1).

### 3.2. Manipal

**Figure 2:**
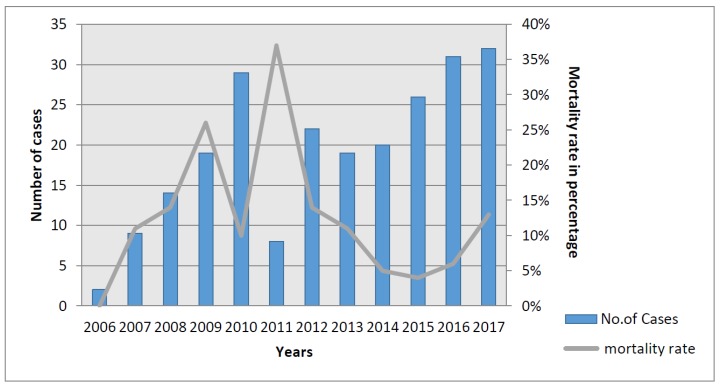
Annual numbers of melioidosis cases and mortality from Manipal.

The tertiary care hospital of Kasturba Medical College in Manipal has more than 2000 beds and receives referred cases from primary and secondary health care centers. The hospital caters to the hilly regions and the plains of Udupi district, and other neighboring districts including Uttar Kannada, Shimoga, Davangere, Chikmagalur, Hassan, and Mangalore with many inpatient admissions (approximately 72,025/year) and outpatient visits (approximately 627,210/year). The greatest number of cases of melioidosis in any single center in India has been reported from Manipal since the first cases were recognized there in 2002 [16]. The incidence of melioidosis in Udupi district over the past three years was equivalent to 0.8/1000 hospital admissions and 1/100,000 population. The numbers have increased significantly since 2006 following the upgrading of laboratory facilities and the implementation of a program to raise awareness among doctors (Figure 2). However, being a tertiary referral center in the sparsely populated coastal Udupi district, most patients visiting this hospital are from middle and high socioeconomic backgrounds and may not have had as extensive exposure to the environment as daily laborers or tenant farmers, meaning that this experience may underestimate the true incidence of the disease in this district.

**Figure 3:**
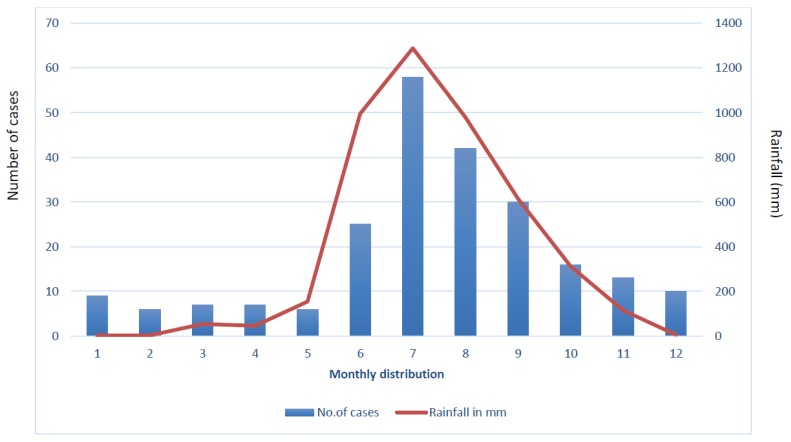
Average monthly rainfall and melioidosis cases in Manipal, 2006 to 2017.

Of the 231 patients with melioidosis diagnosed during 2006–2016 (which includes 82 cases included in the literature review described above), the majority (84, 36.3%) presented with pulmonary infection. Most patients presented during months of heavy rainfall (Figure 3). Organ failure was observed in 43 (19%). Diabetes mellitus was the most common co-morbidity (159, 69%). Overall, 48% of patients were bacteremic, and bacteremia (22/27; 81.4%) along with renal dysfunction (13/27, 48%) were significantly associated with mortality (*p* = 0.004). Mortality appears to have declined over the past decade (Figure 2) and at present the rate is surprisingly low (27, 12%) compared with that in Thailand (35%) [17], and equivalent to that in northern Australia (14%) [18]. In fact, melioidosis causes a relatively small proportion of all deaths attributable to infectious diseases in Manipal. There are no obvious links between changes in clinical management and this low mortality, although it is likely that there has been an increasing awareness over the years, which might have led to more accurate diagnosis and prompt treatment. The socioeconomic status of the patients seeking care at the tertiary center may also mean that they are in better general health than populations in some other settings. However, many patients are discharged against medical advice (DAMA) (mean 300/year over the past four years), mostly due to an inability to afford expensive medical treatment, and their outcome remains unknown. A point prevalence study conducted at Kasturba Hospital (unpublished data) showed that 60% of patients admitted to the hospital with suspected infection received empirical antibiotics that would have been expected to be effective against melioidosis, mainly carbapenems or beta-lactam/beta-lactamase inhibitor combinations. This could also be a cause of these patients remaining undiagnosed by culture as well as having low mortality. It is also likely that there are many patients who seek treatment in primary or secondary health care, private clinics, nursing homes or government hospitals, who remain undiagnosed or misdiagnosed and whose deaths would never be attributed to melioidosis.

### 3.3. Vellore

The Christian Medical College (CMC), Vellore, is a tertiary care teaching hospital with more than 2800 beds, which caters to a wide populace with substantial representation from most states in India, in addition to the patients referred from nearby districts in Tamil Nadu and Andhra Pradesh. The initial reports from CMC helped the recognition of the endemicity of melioidosis in India since 1996 [10]. The most recent retrospective analysis of 114 patients with culture-confirmed melioidosis at CMC between January 2008 and December 2014 reflects the current scenario [19]. Patients came from 15 states in India, but the greatest numbers were residents of West Bengal (26.3%), Jharkhand (22.8%) and Tamil Nadu (14.9%), predominantly from rural areas. As previous studies have shown, diabetes mellitus was found to be the most common risk factor (82.3%). Of the total cases, 41 (36%) presented with acute disease (<2 months duration) while 73 (64%) had chronic manifestations. Patients with acute septicemic melioidosis more frequently had bacteremia (80% vs. 41%; *p* < 0.001) and respiratory involvement (39% vs. 16.4%; *p* = 0.007). The mortality for acute septicemic melioidosis was 17% as opposed to 13.6% among those with chronic disease, with an overall case fatality rate of 14.9%. Respiratory involvement, bacteremia, septic arthritis and skin involvement more commonly occurred with acute melioidosis, while chronic melioidosis had splenic, genitourinary and bone involvement. Respiratory involvement and bacteremia were found to be independent predictors of mortality. Drug susceptibility to carbapenems and ceftazidime was 100%, while resistance to trimethoprim-sulfamethoxazole and doxycycline was found in 5.9% and 2.6%, respectively. Intensive therapy with ceftazidime or meropenem followed by eradication treatment with trimethoprim-sulfamethoxazole, with or without doxycycline, was administered in these patients. Relapse occurred in four (3.5%) patients after periods of 2–7 years. Since the patient population was representative of regions that are geographically distant, this is indicative of the widespread endemicity of the disease within India. However, the numbers are likely to be a substantial underestimate as the diagnosis was only made in patients who were managed at the tertiary care center and many cases are likely to have gone undetected in these regions due to inadequate diagnostic facilities and limited awareness.

### 3.4. Other Neighboring Countries

Only a single case of imported melioidosis has been reported to date from Nepal, in a 35-year-old patient returning from Malaysia [20]. There is some confusion in the literature about cases of melioidosis originating from Pakistan due to the previous designation of Bangladesh as ‘East Pakistan’ [21]. A case of pneumonia was diagnosed in the UK in a 60-year-old male described as being from Pakistan [22]. Inquiries of the author of this report by one of us (DABD) revealed some uncertainty about his true origin, and we have been unable to identify a single case of melioidosis that was unequivocally acquired in Pakistan. No cases were identified from Bhutan or Afghanistan. However, at this stage, it is unclear whether this reflects absence of the disease or simply lack of awareness and diagnostic capability in these countries, as Limmaturotsakul et al. predicted that it is likely that melioidosis exists undiagnosed in Nepal, Pakistan and Bhutan [2].

### 3.5. Serosurveillance

The only serosurveillance study undertaken in India since the 1990s was conducted among 711 healthy residents of Udupi Taluk. The study revealed an overall seropositivity rate (defined as a titer of ≥1:20 in the indirect hemagglutination (IHA) test) of 29% [23]. This suggests that exposure to *B.* *pseudomallei* is relatively common, although cross-reaction with closely-related organisms cannot be excluded [24].

### 3.6. Animals

There have been very few reports of animal melioidosis from any of the South Asian countries. A single case in a male rhesus monkey imported from India was reported from the USA in 1969 [25]. *B. pseudomallei* was also reported to have been associated with two cases of bovine abortion in India, although the antibiogram of the organisms isolated make it very unlikely that these were really *B.* *pseudomallei* [26].

### 3.7. Environmental Evidence

There have been very few studies attempting to isolate *B.* *pseudomallei* from the environment in India. One study in rice paddy along the southeast coast of Tamil Nadu confirmed four environmental isolates of *B. pseudomallei* [27]. Another study conducted along the Malabar coastal region of Kerala found that 22.7% of the *Burkholderia* spp. strains isolated were *B. pseudomallei* [28]. Both studies were conducted at a single time point and used culture followed by 16S rDNA sequencing rather than specific *B. pseudomallei* PCR. In addition, a study from the Lahore district in Pakistan reported the detection of *Burkholderia* spp. genomes in two (1.4%) environmental samples, although the method used could not distinguish between *B*. *pseudomallei* and *B. mallei* [29]. Until further studies are done in South Asia, the true distribution of *B. pseudomallei* in the environment will remain uncertain.

### 3.8. Molecular Epidemiology in South Asian Countries

**Figure 4:**
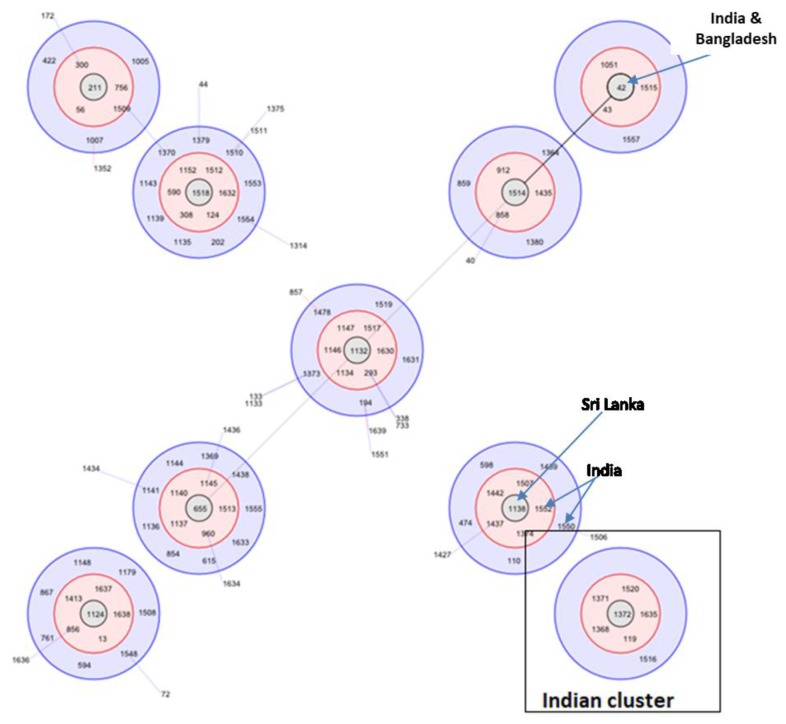
BURST cluster for South Asian countries with red shades depicting single locus variants and blue shades depict double locus variants linking the various STs. A group of Indian STs form a separate cluster not linked to other STs reported from other South Asian countries.

The molecular epidemiology of South Asian isolates of *B. pseudomallei* has not been studied intensively. The first molecular typing of Indian strains revealed several novel sequence types, some of which were single locus variants (SLVs) of isolates from Thailand, Kenya, and China, suggesting the possibility of the spread of *B. pseudomallei* along historical trading routes [30]. A study using multi-locus sequence typing (MLST) of 32 clinical isolates from India found that the majority (93.7%) had novel allelic profiles, with ST 1368 as the most common sequence type [31]. The majority of the isolates were outliers in a population snapshot suggesting that Indian isolates are genetically distinct from Australian and Southeast Asian isolates. However, some of the Indian isolates were found to be single-locus variants of Sri Lankan isolates in the BURST analysis (Figure 4). This is perhaps not surprising, given that Sri Lanka is a neighboring country with which there has always been an active trade of goods and livestock. However, one of the sequence types (STs) reported from the northeastern region of India (ST1051) was similar to an Australian isolate, and homoplasy cannot be ruled out as a cause of such similarities without further molecular analysis such as whole genome sequencing [32].

## 4. Current Recommendations and Availability of Measures against Melioidosis

It is well-established that melioidosis is associated with activities involving exposure to soil and water [33]. Although it is likely that the risk of disease can be reduced by simple measures such as the use of protective gear such as boots and consumption of boiled or bottled water [34], there are no current guidelines implemented in India or other South Asian countries for prevention of melioidosis. The need for enhanced surveillance is discussed below and it is hoped that, as the number of cases detected increases, state and national health authorities will develop and disseminate relevant guidance, particularly targeted at the areas and populations identified to be at greatest risk.

### Surveillance Systems and Reporting

Melioidosis is not currently notifiable in any country in South Asia. In India, for example, diseases like tuberculosis, malaria, and dengue are highly endemic and are covered by specific guidelines and public health policies that have been promulgated by the government to both the public and health workers. The Integrated Disease Surveillance Program (IDSP) run by the Indian National Center for Disease Control (NCDC) encompasses the top 12 priority diseases for laboratory-based surveillance, which include dengue, chikungunya, Japanese encephalitis, meningococcal meningitis, typhoid fever, diphtheria, cholera, shigella dysentery, viral hepatitis (A and E), leptospirosis and malaria (http://idsp.nic.in/index1.php?lang=1&level=1&sublinkid=5985&lid=3925). An additional 22 syndromes are identified for ‘presumptive’ (i.e., clinically-based) surveillance. There are plans to integrate and decentralize surveillance activities at the grassroots level to combat these diseases, along with the development of human resource through training, upgrading of information and communication technology for collection, collation, compilation, analysis and dissemination of data, and strengthening of public health laboratories. However, melioidosis does not come under the surveillance program of NCDC. It has been predicted that the number of deaths from melioidosis globally is higher than that of dengue and leptospirosis [2], both of which are covered by IDSP. If so, the disease would warrant much greater attention. Nonetheless, it is unlikely that this will happen unless surveillance improves and greater numbers of cases are detected, an unfortunate ‘Catch-22’ situation. Currently, the only sources of hard data about melioidosis in South Asia are published case reports, which undoubtedly are still only the tip of the iceberg. It is thus down to South Asian researchers to provide evidence to local policymakers to raise melioidosis up their list of priorities with the ultimate aim of gaining a better understanding of the true burden of the disease through appropriate diagnosis and surveillance.

## 5. Diagnostic Facilities

The clinical features of melioidosis are not characteristic and may be confused with those of many other tropical diseases, especially tuberculosis. Diagnosis of melioidosis thus requires the facilities and expertise of the microbiology laboratory through isolation and identification of *B*. *pseudomallei*, which remains the ‘gold standard’ for diagnosis of the infection. Although the organism is classified as a ‘Tier 1 Select Agent’ in the USA (https://www.selectagents.gov/ohp-app1.html) and is worked on at biosafety level 3 in many countries, such facilities are generally not available in clinical laboratories in South Asian countries, and specimens are usually processed at biosafety level 2. Standard culture media are generally used, although it may take several days to grow the organism. Selective media such as Ashdown’s agar and broth, which increase the yield of *B. pseudomallei* from non-sterile body sites, are rarely available outside a few specialist centers. In Manipal, for example, enrichment with ‘CV-C50’ broth for samples from non-sterile sites has improved the yield of culture [35]. Blood culture is the specimen most frequently received by laboratories for the investigation of melioidosis. Continuously monitored automated blood culture systems have largely replaced conventional blood culture broth or biphasic media in many settings, at least in most private tertiary care hospitals in India. In Manipal, the mean time to positivity for blood culture from patients with bacteremic melioidosis was 30.6 ± 14.2 h (unpublished data). Of 27 patients who died, the blood cultures became positive within 24 h in 21 (77.7%) and 6 (22.2%) within 48 h of the sample collection, results that probably reflect high levels of bacteremia, as has been shown in studies elsewhere [36].

Various methods are used to identify Gram-negative bacilli in South Asian laboratories. With the advent of automated identification systems, most laboratories use methods such as API 20NE and VITEK 2 for identification of the organisms. However, it is likely that many isolates are being dismissed as contaminants without being identified, or labeled, as ‘*Pseudomonas* sp.’ or ‘*Burkholderia* sp.’ *Burkholderia* spp. account for 1–9% of isolates from patients in various multicenter studies of bacteremia in India [37,38,39]. In Manipal, *Burkholderia* spp. were isolated from 1.6% (114/7100) of positive blood cultures over the past six years, of which 68.4% (78/114) were confirmed as *B. pseudomallei* by type III secretion system 1 (TTSS1) PCR. It is unlikely that Manipal is unique, which implies that many cases of melioidosis are probably being missed in places where such isolates are not being correctly identified to species level. In practice, any laboratory in South Asia could make a presumptive identification of an oxidase-positive Gram-negative rod as *B. pseudomallei* using a simple three-antibiotic disc test that was recently shown to be highly specific in Vietnam [40]. Such an approach might usefully be promoted.

New methods are needed to reduce the time to diagnosis and appropriate treatment. One such test, the Active Melioidosis Detect Lateral Flow Immunoassay (AMD-LFA) (InBios Inc., Seattle, WA, USA), has shown promising results as a ‘point-of-care’ (POC) test for early diagnosis of melioidosis [41]. The kit has been evaluated in Manipal on clinical samples from patients suspected of having melioidosis and had a sensitivity of 85.7% and specificity of 93.6% compared with enrichment culture [42]. However, the test gave some weak false-positive results on urine specimens and thus needs further development and evaluation before it can be recommended as a POC test in endemic regions like South Asia.

## 6. Antibiotic Susceptibility and Treatment

Although there are no national guidelines for treating melioidosis in India or other South Asian countries, physicians in centers where cases are regularly diagnosed generally follow international guidelines, i.e., ceftazidime during the acute phase and co-trimoxazole, which is cheap and widely available, for eradication therapy. Generally, the antimicrobial susceptibility of isolates of *B. pseudomallei* in South Asia mirrors that from other regions [43], although rare instances of ceftazidime resistance have been reported [44]. Clinical failure of treatment despite apparent in vitro susceptibility has also been described [45].

## 7. Awareness of Melioidosis

There is an overall lack of awareness of melioidosis in South Asian countries, as is even the case in countries where the disease is diagnosed more often, such as Thailand [46]. Although there have been no formal surveys of knowledge and understanding about the disease, our day-to-day observations reveal that most doctors outside a few specialist centers have never heard of it, the public is completely unaware of it, and it is not on the radar of policymakers, either at a state or a national level.

## 8. Establishing a Melioidosis Network

**Figure 5:**
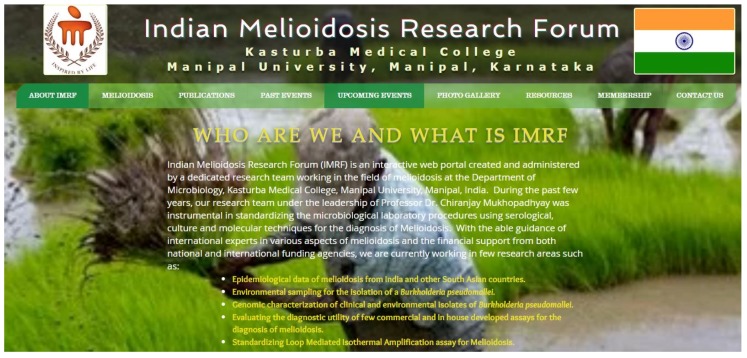
The Indian Melioidosis Research Forum (IMRF) has effectively brought researchers from all over India for research in melioidosis for the last two years.

The Indian Melioidosis Research Forum (IMRF; www.melioidosisindia.com—Figure 5) was initiated in 2015 during the First South Asian Melioidosis Congress (1st SAMC) in Manipal as an interactive web portal intended to enhance communication between those in South Asia with an interest in melioidosis. Thus far, it has connected researchers from 12 states of India and encouraged research on melioidosis. An earlier workshop about laboratory processing and environmental sampling had been conducted in Manipal in collaboration with the University of Cambridge, UK, and Mahidol University, Thailand. Seven centers have already started detecting cases of melioidosis in their own states after becoming involved in the network and are in regular contact with IMRF regarding the diagnosis of the disease and confirmation of the isolates. IMRF is well connected with the International Melioidosis Network (www.melioidosis.info) and is in a position to act as a regional leader to connect enthusiastic researchers from South Asia with the international melioidosis community. The ‘Center for Emerging and Tropical Diseases’ in Manipal was established in 2017 and now acts as an informal reference laboratory for melioidosis. To date, it has confirmed 80 clinical isolates of *B. pseudomallei* from laboratories in different states of India, including Orissa, Hyderabad, Kerala, Pondicherry, and Assam (Table 2). The center is currently working with the Medical University of Graz, Austria (Indo-Austrian collaboration) and the Central Soil Salinity Research Institute (CSSRI), Haryana, on the ecology of *B. pseudomallei*, and the Defense Research and Development Organization (DRDO), Gwalior, on the molecular epidemiology of the pathogen. It has been instrumental in mentoring researchers and microbiologists around the country and encouraging them to share their expertise and collaborate in future projects.

## 9. Current and Future Challenges

Melioidosis is clearly an emerging disease in South Asia. The number of cases that have been microbiologically confirmed is still relatively small and it is too early to know whether the huge numbers of cases predicted to occur each year (more than 50,000 cases and 30,000 deaths in India alone) are anywhere near accurate. However, India faces an ‘outbreak’ of diabetes of enormous proportion. The prevalence of diabetes in India, according to the IDF Diabetes Atlas 2015 (http://www.diabetesatlas.org/across-the-globe.html), is reported to be 8.7% and, given the strong association between diabetes and melioidosis, it is likely that the latter will become more common as the former increases. Sadly, diagnostic microbiology services and laboratory-based surveillance across South Asia remain poorly developed, particularly those serving the rural poor, which is the very group that is at greatest risk of melioidosis.

The key challenges facing those with an interest in melioidosis in South Asia at this stage are as follows:

1. Collecting and collating better data on the number of cases of melioidosis occurring will require extensive communication with laboratories that are capable of identifying the organism.
2. Training more physicians and laboratory staff to recognize the clinical features of the disease and the characteristics of its causative organism (for example, through the use of the three-antibiotic disc test mentioned above) will require concerted efforts through universities and colleges and professional associations but will be difficult because of the fragmented nature of healthcare in the region.
3. Raising the profile of the disease with both policymakers and the general public at both local and national levels will inevitably have to be started locally, but, as the network of those with an interest grows, it should become easier to provide the necessary evidence that the morbidity and mortality of the disease warrant greater attention. One key objective would be to have melioidosis included in the list of diseases that are statutorily reportable by laboratories, which in turn would improve knowledge of the disease distribution and burden.

## 10. Conclusions

The burden of melioidosis in South Asia is predicted to be considerable, but as yet the evidence to support this is scant. However, given the huge population, the very high prevalence of underlying conditions predisposing to melioidosis such as diabetes mellitus, the shortcoming of diagnostic laboratory services, and the lack of awareness amongst health professionals, it remains possible that the predictions could indeed prove to be correct. There is evidence that interest in the disease is growing amongst healthcare workers and researchers, but the key challenge will be to coordinate efforts across this vast and diverse region. It is hoped that initiatives such as the IMRF and the SAMC (the second of which was held in Sri Lanka in 2017 and the next will take place in Bangladesh in 2020) will help to achieve this.

## Figures and Tables

**Table:** 

Month	Season
October–February	Post-monsoon
March–May	Summer
June–September	Monsoon

**Table 1:** Published cases of melioidosis reported from different states of India (1991–2018) (see Supplementary material for reference list).

State	Number of Cases	Age Range	Gender (Male: Female)	Diabetes n (%)	Intensive Treatment		Eradication Treatment		Mortality n (%)
					MER	CAZ	SXT	Others	
Karnataka	306	0–84	3:1	241 (79)	24	294	235	50	39 (13)
Tamil Nadu	146	4–65	3:1	75 (50)	3	82	80	2	37 (28)
Telangana	35	30–66	2:1	27 (77)	8	7	11	3	4 (11)
Kerala	34	9–66	5:1	24 (77)	3	15	14	2	5 (13)
Pondicherry	14	0–58	3:1	3 (21)	2	2	-	4	2 (14)
West Bengal	11	29–71	11:0	9 (82)	6	4	9	1	2 (18)
Maharashtra	9	10–72	7:1	5 (50)	0	4	4	0	4 (50)
Orissa	8	47–51	7:0	5 (63)	0	6	5	0	1 (12.5)
Assam	6	0–57	2:1	3 (50)	3	0	1	1	2 (33)
Goa	5	34–53	5:0	5 (100)	2	2	1	1	0
Bihar	4	50–65	4:0	4 (100)	2	1	2	1	0
Jharkhand	2	32–33	2:0	1 (50)	1	1	1	0	1 (50)
Rajasthan	1	49	1:0	0	1	0	1	0	0
Madhya Pradesh	1	56	1:0	1 (100)	0	1	0	0	0
Andhra Pradesh	1	23	1:0	1 (100)	0	0	0	0	1 (100)

Abbreviations: MER, meropenem; CAZ, ceftazidime; SXT, cotrimoxazole. Details are not given in many publications so denominators differ in each column.

**Table 2:** Isolates received by the Manipal Center for Emerging and Tropical Diseases for confirmation from various centers in India.

Institute (State)	Number of Isolates
Amritha Institute of Medical Sciences (Kerala)	34
Pondicherry Institute of Medical Sciences (Puducherry)	20
Nizams Institute of Medical Sciences (Hyderabad, Telangana)	12
Government Medical College (Tiruvanthapuram, Kerala)	7
Kalinga Institute of Medical Sciences (Orissa)	4
Government Medical College and Hospital (Mallapuram, Kerala)	2
Tripura Medical College (Assam)	1

## Supplementary Materials

They are available online at http://www.mdpi.com/2414-6366/3/2/51/s1.
