# Supplementary material for: Melioidosis in South Asia (India, Nepal, Pakistan, Bhutan and Afghanistan)
Source: Trop Med Infect Dis. 2018 May 22;3(2):51. doi: 10.3390/tropicalmed3020051 (PMC6073985; doi:10.3390/tropicalmed3020051)
Supplement: Supplementary file 1 [file tropicalmed-03-00051-s001.pdf]

Supplementary File 1

Melioidosis – India References

1. Abdulla MC, Alungal J. Melioidosis with endocarditis and massive cerebral infarct. Ital J Med. 2016;10(1):55-7.
2. Achappa B, Madi D, Vidyalakshmi K. Cutaneous melioidosis. J ClinDiagn Res. 2016;10(9):WD01-WD2.
3. Agrawal R, Sharma D, Dhiman P, Patro DK. Clinical and haematological predictors of acute hematogenous Methicillin Resistant *Staphylococcus aureus* (MRSA) osteomyelitis & septic arthritis. Journal of Orthopaedics. 2015;12(3):137-41.
4. Al Alousi FS, Al Soub H, El-Shafie SS. Subdural empyema due to *Burkholderia pseudomallei*. Annals of Saudi Medicine. 2000;20(3-4):272-3.
5. Ali HM, George P. Concurrent abscesses of lung and orbit due to *Bukholderia pseudomallei* : A rarity. Journal of the Association of Physicians of India. 2016;64(1):55-6.
6. Amrutha M, Rajagopal TP. Cutaneous melioidosis. Quart J Med. 2016;109(2):129.
7. Anandan S, Augustine A, Mathai E, Jesudason MV. Evaluation of IgM ELISA using a sonicate and a lipopolysaccharide antigen for the serodiagnosis of melioidosis. Indian Journal of Medical Microbiology. 2010;28(2):158-61.
8. Anandraj VK, Priyadharshini A, Sunil SS, Ambedkar RK. *Burkholderia pseudomallei* infection in a healthy adult from a rural area of South India. Indian Journal of Pathology& Microbiology. 2012;55(4):578-9.
9. Antony B, Pinto H, Dias M, Shetty AK, Scaria B, Kuruvilla T, et al. Spectrum of melioidosis in the suburbs of Mangalore, S West Coast of India. The Southeast Asian Journal of Tropical Medicine and Public Health. 2010;41(1):169-74.
10. Antony T, Moorthy S, Narayanaswamy A, Arthur P. Melioidosis presenting as septicaemia and facial nerve palsy. BMJ Case Reports. 2017;2017:04.
11. Anuradha K, Meena AK, Lakshmi V. Isolation of *Burkholderia pseudomallei* from a case of septicaemia--a case report. Indian Journal of Medical Microbiology. 2003;21(2):129-32.
12. Arockiaraj J, Karthik R, Jeyaraj V, Amritanand R, Krishnan V, David KS, et al. Non-Caseating Granulomatous Infective Spondylitis: Melioidotic Spondylitis. Asian Spine J. 2016;10(6):1065-71.
13. Arora S, Thavaselvam D, Kumar A, Prakash A, Barua A, Sathyaseelan K. Cloning, expression and purification of outer membrane protein (OmpA) of *Burkholderia pseudomallei* and evaluation of its potential for serodiagnosis of melioidosis. Diagnostic Microbiology and Infectious Disease. 2015;81(2):79-84.
14. Balaji V, Jesudason MV, Sridharan G, Subramanian K. Detection of virulence attributes of *Burkholderia pseudomallei*. The Indian Journal of Medical Research. 2004;119(3):101-6.
15. Balaji V, Rajiv K, Abraham OC. *Burkholderia pseudomallei* recovered in an HIV-positive individual. Indian Journal of Medical Sciences. 2008;62(11):456-8.

16. Barman P, Kaur R, Kumar K. Clinically lesser known entity in India: A Report of two cases of Melioidosis. Indian Journal of Critical Care Medicine. 2013;17(1):46-8.
17. Barman P, Sidhwa H, Shirkhande PA. Melioidosis: a case report. J Glob Infect Dis. 2011;3(2):183-
18. Behera B, Mohanty S, Mishra B, Praharaj AK. Melioidosis: An underdiagnosed entity in Odisha. A series of four cases over a two months period. Int J Infect Dis. 2016;45:166-.
19. Behera B, Prasad Babu TL, Kamalesh A, Reddy G. Ceftazidime resistance in *Burkholderia pseudomallei*: first report from India. Asian Pac J Trop Med. 2012;5(4):329-30.
20. Bharadwaj R, Kagal A, Deshpandey SK, Joshi SA, Khare PM, Junnarkar AR, et al. Outbreak of plague-like illness caused by *Pseudomonas pseudomallei* in Maharashtra, India. Lancet. 1994;344(8936):1574.
21. Bharadwaj R, Kagal A, Deshpandey SK, Joshi SA, Khare PM, Junnarkar AR, et al. *Burkholderia pseudomallei* and Indian plague-like illness.[Erratum appears in Lancet 1995 Oct 28;346(8983):1172]. Lancet. 1995;346(8980):975.
22. Bharranitharan., Subashini S, Durai R. Pyogenic sacroilitis due to *Burkholderia pseudomallei* resulting in septic shock: a case report. Journal of Medical Science and Clinical Research. 2017;05(02):18018-20.
23. Bommakanti K, Ankathi P, Uma P, Malladi S, Laxmi V. Cerebral abscess and calvarial osteomyelitis due to *Burkholderia pseudomallei*. Neurology India. 2010;58(5):801-2.
24. Boruah DK, Prakash A, Bora R, Buragohain L. Acute pulmonary melioidosis in a child: A case report and review of literature. Indian Journal of Radiology and Imaging. 2013;23(4):310-2.
25. Chandrakar S, Dias M. Clinico-epidemiological spectrum of melioidosis: a 2-year prospective study in the western coastal region of India. Southern African Journal of Infectious Diseases. 2016;31(1):14-9.
26. Chandrakar S, Dias M. Soil sampling of *Burkholderia pseudomallei* in and around Mangalore, India - an explorative study. Journal of Bacteriology and Mycology. 2017;4(2):1046.
27. Charles MV, Easow JM, Joseph NM, Ravishankar M, Kumar S, Umadevi S. Role of Appropriate Therapy in Combating Mortality among the Ventilated Patients. Journal of Clinical and Diagnostic Research : JCDR. 2014;8(8):DC01-3.
28. Cheng AC, Johnson DF. Multi loculated hepatosplenic abscesses. Clinical Infectious Diseases. 2006;43(2):264-5.
29. Cherian T, John TJ, Ramakrishna B, Lalitha MK, Raghupathy P. Disseminated melioidosis. Indian Pediatrics. 1996;33(5):403-6.
30. Cherian T, Raghupathy P, John TJ. Plague in India. Lancet. 1995;345(8944):258-9.
31. Chrispal A, Rajan SJ, Sathyendra S. The clinical profile and predictors of mortality in patients with melioidosis in South India. Tropical Doctor. 2010;40(1):36-8.
32. Chugh TD. Emerging and re-emerging bacterial diseases in India. Journal of Biosciences. 2008;33(4):549-55.
33. Cousins S. India is at high risk from surge in cases of melioidosis, warn researchers. BMJ. 2016;352:i275.

34. Dance DAB, Sanders D, Pitt TL, Speller DC. *Burkholderia pseudomallei* and Indian plague-like illness. Lancet. 1995;346(8979):904-5.

35. Danda D, Thomas K. Transient asceptic arthritis of knees in a patient with melioidosis. Indian Journal of Medical Sciences. 2000;54(1):18-20.

36. Das AM, Paranjape VL. *Pseudomonas pseudomallei* associated with bovine abortion in India. Veterinary Medical Review. 1986;2:173-6.

37. Deshmukh M, Mundhada S. Chronic suppurative joint effusion due to *Burkholderia pseudomallei*: a case report. Indian Journal of Pathology & Microbiology. 2013;56(4):460-3.

38. Dhodapkar R, Sujatha S, Sivasangeetha K, Prasanth G, Parija SC. *Burkholderia pseudomallei* infection in a patient with diabetes presenting with multiple splenic abscesses and abscess in the foot: a case report. Cases Journal. 2008;1(1):224.

39. Dias E, Dias M. Systemic melioidosis. Indian Pediatrics. 2011;48:161.

40. Dias M, Antony B, Aithala S, Hanumanthappa B, Pinto H, Rekha B. *Burkholderia pseudomallei* septicaemia - a case report. Indian Journal of Medical Microbiology. 2004;22(4):266-8.

41. Ekka AS, Mohideen M, Kesavan S. Neuromelioidosis masquerading as acute demyelinating encephalomyelitis. Indian Pediatrics. 2017;54(12):1054-5.

42. Gandra S, Mojica N, Klein EY, Ashok A, Nerurkar V, Kumari M, et al. Trends in antibiotic resistance among major bacterial pathogens isolated from blood cultures tested at a large private laboratory network in India, 2008–2014. Int J Infect Dis. 2016;50:75-82.

43. Garg G, Chawla N, Chawla K, Khosla P, Jain S. Atypical presentations of melioidosis in North India: Report of two cases. J AssocPhys India. 2015;63(JUNE):82-3.

44. Garg R, Shaw T, Bhat SN, Mukhopadhyay C. Melioidosis: The great mimicker presenting as spondylodiscitis. BMJ case reports. 2018;2018.

45. Goel A, Bansal R, Sharma S, Singhal S, Kumar A. Chronic melioidosis presenting with multiple abscesses. Oxf Med Case Reports. 2016;2016(6):113-6.

46. Gopalakrishnan R, Sureshkumar D, Thirunarayan MA, Ramasubramanian V. Melioidosis: an emerging infection in India. The Journal of the Association of Physicians of India. 2013;61(9):612-4.

47. Gouse M, Jayasankar V, Patole S, Veeraraghavan B, Nithyananth M. Clinical Outcomes in Musculoskeletal Involvement of *Burkholderia pseudomallei* Infection. ClinOrthop Surg. 2017;9(3):386-91.

48. Gundapuneni N, Vani J, Talluri SC. Disseminated melioidosis involving clavicular bone - A rare case report. Journal of the Association of Physicians of India. 2016;64(1):119.

49. Halim I, Shaw T, Tellapragada C, Vandana KE, Mukhopadhyay C. Melioidosis: Reinfection going incognito as relapse. Indian Journal of Medical Microbiology. 2017;35(4):593-6.

50. Handa R, Bhatia S, Wali JP. Melioidosis: a rare but not forgotten cause of fever of unknown origin. British Journal of Clinical Practice. 1996;50(2):116-7.

51. Handa R, Wali JP. Melioidosis in India. Journal of the Association of Physicians of India. 1996;44(7):510.

52. Ives JC, Thomson TJ. Chronic melioidosis: the first report of a case infected in central India. Glasgow Medical Journal. 1953;34(2):61-7.

53. Jagtap N, Shah H, Kancharla A, Tandan M, Pal P, Lakhtakia S, et al. Gastrointestinal manifestations of melioidosis: A single center experience. Indian J Gastroenterol. 2017:1-4.
54. Jain VK, Jain D, Kataria, H, Shukla A, Arya RK, Mittal D. Melioidosis: A review of orthopedic manifestations, clinical features, diagnosis and management. Indian Journal of Medical Sciences. 2007;61(10):580-90.
55. Jakribettu R, Boloor R, D'Souza R, Aithala S. Subcutaneous surprise. Annals of Medical and Health Sciences Research. 2014;4(1):123-5.
56. James D, Madhuri V, Gahukamble AD, Choudhrie L, Pancharatnam P. *Burkholderia pseudomallei* osteomyelitis of the metatarsal in an infant. The Journal of Foot and Ankle Surgery 2013;52(3):370-3.
57. Jamkhandi DM, Alex R, George K. Melioidosis: A report of two cases. Natl Med J India. 2014;27(4):202-3.
58. Jayakumar E, Barani R, Mani M, Seshan V, Kothandaramanujam SM, Balakrishnan R, et al. Molecular evidence of melioidosis among patients suspected for tuberculosis. Int J Infect Dis. 2016;45:34.
59. Jaya S, Harita V. Melioidosis - cases with various clinical presentations: a case report. International Journal of Scientific Research. 2014;3(4):324-5.
60. Jayaprakash B, Karthik Rao N, Patil N, Balaji O, Rau NR, Varghese G. Melioidosis: A rare case of hemoptysis with pseudoaneurysm. Res J Pharm, Biol Chem Sci. 2016;7(3):1977-81
61. Jesudason M, Shanthakumari R, John TJ. *Burkholderia pseudomallei* - An Emerging Pathogen in India. Indian Journal of Medical Microbiology. 1997;15(1):1-2.
62. Jesudason MV, Anandaraj WS, Malathi B. An indirect ELISA for the diagnosis of melioidosis. The Indian Journal of Medical Research. 2001;114:51-3.
63. Jesudason MV, Anbarasu A, John TJ. Septicaemic melioidosis in a tertiary care hospital in south India. The Indian Journal of Medical Research. 2003;117:119-21.
64. Jesudason MV, Balaji V, Sirisinha S, Sridharan G. Rapid identification of *Burkholderia pseudomallei* in blood culture supernatants by a coagglutination assay. Clinical Microbiology and Infection 2005;11(11):930-3.
65. John TJ. Final thoughts on India's 1994 plague outbreaks. Lancet. 1995;346:765.
66. John TJ. Emerging & re-emerging bacterial pathogens in India. The Indian Journal of Medical Research. 1996;103:4-18.
67. John TJ. Melioidosis, the mimicker of maladies. The Indian Journal of Medical Research. 2004;119(3):vi-viii.
68. John TJ, Jesudason MV, Lalitha MK, Ganesh A, Mohandas V, Cherian T, et al. Melioidosis in India: the tip of the iceberg? The Indian Journal of Medical Research. 1996;103:62-5.
69. Kamath MP, Bhojwani K, Chakrapani M, Vidyalakshmi KP, Vishnuprasad KP. Melioidosis of salivary glands with coexisting diabetes: management of a difficult case. Ear, Nose, & Throat Journal. 2014;93(1):E22-5.
70. Kang G, Rajan DP, Ramakrishna BS, Aucken HM, Dance DAB. Melioidosis in India. Lancet. 1996;347(9014):1565-6.
71. Kanungo R, Padhan P, Bhattacharya S, Srimannarayana J, Jayanthi S, Swaminathan RP. Melioidosis--a report from Pondicherry, South India. Journal of the Association of Physicians of India. 2002;50:1438-9.

72. Karanth SS, Regunath H, Chawla K, Prabhu M. A rare case of community acquired Burkholderiacepacia infection presenting as pyopneumothorax in an immunocompetent individual. Asian Pac. 2012;2(2):166-8.
73. Karthik R, Pancharatnam P, Balaji V. Fatal *Chromobacterium violaceum* septicemia in a South Indian adult. Journal of Infection in Developing Countries. 2012;6(10):751-5.
74. Karuna T, Khadanga S, Dugar D. Melioidosis: Indian perspective. International Journal of Medical Research and Review. 2014;2(3):243-8.
75. Karuna T, Khadanga S, Dugar D, Sau B, Bhoi P. Melioidosis as a cause of acute abdomen in immuno-competent male from eastern India. J. 2015;7(1):58-60.
76. Khadanga S, Karuna T, Dugar D, Satapathy SP. *Chromobacterium violaceum*- induced sepsis and multiorgan dysfunction, resembling melioidosis in an elderly diabetic patient: A case report with review of literature. J Lab Physicians. 2017;9(4):325-8.
77. Krishnan P, Fernandes S, Savio J, Ross CR, Pradeep R, Choudhary R, et al. Melioidosis. The Journal of the Association of Physicians of India. 2008;56(AUG):636-9.
78. Krovvidi R, Mridula RK, Jabeen SA, Meena AK. Guillain Barre syndrome as a manifestation of neurological melioidosis. Annals of Indian Academy of Neurology. 2013;16(4):681-3.
79. Kulkarni RD, Jain P, Ajantha GS, Shetty J, Chunchanur S, Shubhada C. Fatal *Burkholderia pseudomallei* septicaemia in a patient with diabetes. The Indian Journal of Medical Research. 2010;131:584-5.
80. Kumar GS, Raj PM, Chacko G, Lalitha MK, Chacko AG, Rajshekhar V. Cranial melioidosis presenting as a mass lesion or osteomyelitis. Journal of Neurosurgery. 2008;108(2):243-7.
81. Kundangar RS, Bhat SN, Mohanty SP. Melioidosis mimicking tubercular cold abscess. BMJ case Reports. 2017;19:19.
82. Kunnathuparambil SG, Sathar SA, Tank DC, Sreesh S, Mukunda M, Narayanan P, et al. Splenic abscess due to chronic melioidosis in a patient previously misdiagnosed as tuberculosis. Annals of Gastroenterology. 2013;26(1):77-9.
83. Kuruvilla TS, Dias M, Udayan U, Furtado Z. Melioidotic pericardial effusion. Indian Journal of Medical Sciences. 2010;64(2):94-8.
84. Lakshmi Prasad G, Kini P, S D. Central nervous system melioidosis in the pediatric age group: review. Childs Nervous System 2017.
85. Lakshmi V, Umabala P, Anuradha K, Padmaja K, Padmasree C, Rajesh A, et al. Microbiological spectrum of brain abscess at a tertiary care hospital in South India: 24-year data and review. Pathology Research International. 2011;2011:583139.
86. Lath R, Rajshekhar V, George V. Brain abscess as the presenting feature of melioidosis. BrJNeuro. 1998;12(2):170-2
87. Loveleena, Chaudhry R, Dhawan B. Melioidosis; the remarkable imitator: recent perspectives. The Journal of the Association of Physicians of India. 2004;52:417-20.
88. Madi D, Rai SP, Vidyalakshmi K, Chowta KN. Neurological melioidosis presenting as intracranial abscess. Indian journal of pathology & microbiology. 2016;59(3):417-9.

89. Malladi SVS, Vemu L, Chandra NC, Shetty M, Adiraju KP, Modugu NR, et al. Clinical spectrum of melioidosis at a tertiary care hospital in South India. Int J Infect Dis. 2016;45:470-.

90. Mamtora DK, Davis AM, Koppikar M, Cunha N, Bhalekar P. *Burkholderia pseudomallei*: Liver abscess in a diabetic patient from Western India. J Clin Diagn Res. 2018;12(3):DD06-DD7.

91. Manasi R, Payyappilly RJ. Study of incidence of melioidosis for a period of two years in a tertiary care hospital in North Kerala, South India. Journal of Medical Science and Clinical Research. 2017;05(06):23057-61.

92. Mathai E, Jesudason MV, Anbarasu A. Indirect immunofluorescent antibody test for the rapid diagnosis of melioidosis. The Indian journal of medical research. 2003;118:68-70.

93. Mathai KR, Bhat KS, Ashraf M, Sarawag M, K PK. Melioidosis with a Pericardial Effusion, which Relapsed as a Chest Wall Abscess: A Rare Presentation. Journal of clinical and diagnostic research : JCDR. 2013;7(4):746-8.

94. Mathew S, Perakath B, Mathew G, Sitaram V, Nair A, Lalitha MK, et al. Surgical presentation of melioidosis in India. Natl Med J India. 1999;12(2):59-61.

95. Miraclin A, Mani S, Suresh S, Iyyadurai R. Septicemic melioidosis with ruptured splenic abscess in a patient with thalassemia intermedia. J Global Infect Dis. 2017;9(1):32-3.

96. Mohamad NI, Harun A, Hasan H, Deris ZZ. In-Vitro Activity of Doxycycline and β-Lactam Combinations Against Different Strains of Burkholderiapseudomallei. Indian journal of microbiology. 2018.

97. Mohanakannan S, Mahankali S. Emerging trends in melioidosis - A case series. Journal of the Association of Physicians of India. 2016;64(1):121.

98. Mohanty S, Pradhan G, Panigrahi MK, Mohapatra PR, Mishra B. A case of systemic melioidosis: unravelling the etiology of chronic unexplained fever with multiple presentations. PneumonolAlergol Pol. 2016;84(2):121-5.

99. Moore TD, Allen AM, Palmer AE. Melioidosis - Maryland. Morbidity and Mortality Weekly Record. 1969;18(32):278-9.

100. Mukhopadhya A, Balaji V, Jesudason MV, Amte A, Jeyamani R, Kurian G. Isolated liver abscesses in melioidosis. Indian journal of medical microbiology. 2007;25(2):150-1.

101. Mukhopadhyay C. Melioidosisendemicity in India. Int J Infect Dis. 2016;45:29-.

102. Mukhopadhyay C, Chawla K, Krishna S, Nagalakshmi N, Rao SP, Bairy I. Emergence of Burkholderiapseudomallei and pandrug-resistant non-fermenters from southern Karnataka, India. Transactions of the Royal Society of Tropical Medicine and Hygiene. 2008;102 Suppl1:S12-7.

103. Mukhopadhyay C, Chawla K, Vandana KE, Krishna S, Saravu K. Pulmonary melioidosis in febrile neutropenia: the rare and deadly duet. Tropical doctor. 2010;40(3):165-6.

104. Mukhopadhyay C, Dey A, Bairy I. Atypical presentations of melioidosis as emerging threat: a case report. Indian journal of pathology & microbiology. 2007;50(4):933-6.

105. Mukhopadhyay C, Eshwara V, Hattangadi V. Melioidosis. Journal of The Academy of Clinical Microbiologists. 2013;15(1):11-8.

106. Mukhopadhyay C, Eshwara VK, Kini P, Bhat V. Pediatric Melioidosis in Southern India. Indian pediatrics. 2015;52(8):711-2.

107. Mukhopadhyay C, Kaestli M, Vandana KE, Sushma K, Mayo M, Richardson L, et al. Molecular characterization of clinical Burkholderiapseudomallei isolates from India. The American journal of tropical medicine and hygiene. 2011;85(1):121-3.
108. Mukhopadhyay C, Vandana KE, Chaitanya TA, Shaw T, Bhat HV, Chakrabarty S, et al. Genome Sequence of a Burkholderiapseudomallei Clinical Isolate from a Patient with Community-Acquired Pneumonia and Septicemia. Genome announcements. 2015;3(4).
109. Mukhopadhyay C, Vandana KE, Krishna S, Saravu K, Shastri BA. Aquatic to pulmonary: Severe melioidosis following near-drowning from Southern India. Internet Journal of Pulmonary Medicine. 2010;11(2).
110. Murali R, Ganesh A, Jesudason M, Mathew RK, Date A. Surat '94: was it melioidosis? - Interesting observations from the first case of imported melioidosis in India. Journal of the Association of Physicians of India. 1996;44(3):218-9.
111. Naganathan K, Pillai SB, Kumar P, Hegde P. Whitmore's disease: an uncommon urological presentation. BMJ case reports. 2014;2014.
112. Nagoba BS, Deshpande JJ. Plague in India. Lancet. 1995;345(8944):259.
113. Naha K, Shastry BA, Saravu K. Colonization or spontaneous resolution: expanding the role for Burkholderiapseudomallei. Asian Pac J Trop Med. 2014;7(3):250-2.
114. Nandagopal B, Sankar S, Lingesan K, Appu K, Sridharan G, Gopinathan A. Application of polymerase chain reaction to detect *Burkholderia pseudomallei* and *Brucella* species in buffy coat from patients with febrile illness among rural and peri-urban population. J Glob Infect Dis. 2012;4(1):31-7.
115. Navaneethan U, Ramesh Kumar AC, Ravi G. Multiple visceral abscess in a case of melioidosis. Indian journal of medical sciences. 2006;60(2):68-70.
116. Nayar SA, Lancy J. Melioidosis- case series in a tertiary care centre in South Kerala. International Journal of Scientific Research. 2017;6(5):378-80.

117. Neliyathodi S, Thazhathethil AN, Pallivalappil L, Balakrishnan D. Pleuropulmonarymelioidosis with osteomyelitis rib. Lung India. 2015;32(1):67-9.
118. Nivedhana S, Rajendran S. Neonatal Melioidosis with Pneumatoceles. Indian pediatrics. 2016;53(4):352.
119. Noyal MJ, Harish BN, Bhat V, Parija SC. Neonatal melioidosis: a case report from India. Indian journal of medical microbiology. 2009;27(3):260-3.

120. Oommen S, Sivan Pillai PM, Viswanathan P, Nair S, Nair K. *Burkholderia pseudomallei*: Three cases in 6 months in Central Travancore. Journal of The Academy of Clinical Microbiologists. 2013;15(1):19-21.
121. P Lakshmi V, K Sai Leela.Melioidosis in Andrha Pradesh,India. International Journal of Scientific Research. 2014;3(10):453-4.
122. Padmaja K, Lakshmi V, Sudhaharan S, Venkata Surya Malladi S, Gopal P, VenkataRavinuthala K. Unusual Presentation of Melioidosis in a Case of Pseudoaneurysm of Descending Thoracic Aorta: Review of Two Case Reports. Res. 2015;4(2):e27205.

123. Pal P, Ray S, Moulick A, Dey S, Jana A, Banerjee K. Liver abscess caused by *Burkholderia pseudomallei* in a young man: A case report and review of literature. World journal of clinical cases. 2014;2(10):604-7.
124. Pandey V, Rao SP, Rao S, Acharya KK, Chhabra SS. *Burkholderia pseudomallei* musculoskeletal infections (melioidosis) in India. Indian journal of orthopaedics. 2010;44(2):216-20.
125. Paneth N. Plague in India. Lancet. 1995;345(8944):258.
126. Patil HG, Gundavda M, Shetty V, Soman R, Rodriques C, Agashe VM. Musculoskeletal melioidosis: An under-diagnosed entity in developing countries. Journal of orthopaedics. 2016;13(1):40-2.
127. Patil NA, Balaji O, Rao KN, Hande HM, Ahmed T, Singhal S. A rare cause of septic arthritis with pleural effusion: *Burkholderia pseudomallei*. Asian J Pharm Clin Res. 2017;10(1):8-9.
128. Peddayelachagiri BV, Paul S, Gogoi M, Sripathy MH, Batra HV. Evaluation of fimC and bdha based duplex PCR for specific identification and differentiation of *Burkholderia pseudomallei* from near-neighbor *Burkholderia* species. International journal of medical microbiology : IJMM. 2017.
129. Pillai MP, Faizal BP, Urs VD. Emerging drug resistance in melioidosis. Ann Trop Med Public Health. 2014;7(6):263-5.
130. Prakash A, Arora S, Thavaselvam D, Kumar A, Barua A, Sathyaseelan K. Evaluation of antibodies raised against native and recombinant antigens in plate elisa format for the immunological characterization of *Burkholderia pseudomallei* causative organism of melioidosis. Asian J MicrobiolBiotechnol Environ Sci. 2014;16(1):121-9.
131. Prasad S, Mitreyee R, Karthik Rao N, Belle J. An unusual site for melioidosis: parotid gland. International Journal of Scientific Research. 2015;4(11):397-8.

132. Prasad G, Nair R, Menon G. Intracranial melioidosis: First report in a human immunodeficiency virus positive individual manifesting as cranial osteomyelitis. Neurology India. 2017;65(6):1423-6.
133. Prasad GL, Kini P, Divya S. Central nervous system melioidosis in the pediatric age group: review. Childs Nervous System. 2017;33(6):1-6.
134. Prasanna Kumar M, Krishnamurthy S, Venkateswaran VS, Mahadevan S, Lalitha M, Sistla S, et al. Brainstem micro-abscesses caused by Burkholderiapseudomallei in a 10-month-old infant: a case report. PaediatrInt Child Health. 2017;37(3):230-2.
135. Pravin Charles MV, Easow JM, Joseph NM, Ravishankar M, Kumar S, Umadevi S. Aetiological agents of ventilator-associated pneumonia and its resistance pattern - A threat for treatment. Australasian Medical Journal. 2013;6(9):430-4.
136. Princess I, Ebenezer R, Ramakrishnan N, Daniel AK, Nandini S, Thirunarayan MA. Melioidosis: An emerging infection with fatal outcomes. Indian J Crit Care Med. 2017;21(6):397-400.
137. Raghavan KR, Shenoi RP, Zaer F, Aiyer R, Ramamoorthy P, Mehta MN. Melioidosis in India. Indian pediatrics. 1991;28(2):184-8.
138. Rajadhyaksha A, Sonawale A, Khare S, Kalal C, Jankar R. Disseminated melioidosis presenting as septic arthritis. The Journal of the Association of Physicians of India. 2012;60:44-5.

139. Rajinikanth J, Balaji V, Gaikwad P, Muthusami JC. Melioidosis of the parotid: the tip of the iceberg. Otolaryngology--head and neck surgery : official journal of American Academy of Otolaryngology-Head and Neck Surgery. 2008;139(5):731-2.
140. Ramamoorthi K, Saravu K, Mukhyopadhyaya C, Barakur AS. Melioidosis: An underdiagnosed disease in India (epidemiology, clinical features, and outcomes). Asian Biomedicine. 2013;7(2):249-56.
141. Rao PS, Dhawan R, Shivananda PG. Burkholderiapseudomallei infections. Tropical doctor. 2002;32(3):174-5.
142. Rao PS, Shivananda PG. Burkholderiapseudomallei--abscess in an unusual site. Indian journal of pathology & microbiology. 1999;42(4):493-4.
143. Ray U, Dutta S, Ramasubban S, Sen D, Tiwary IK. Melioidosis: Series of eight cases. J AssocPhys India. 2016;64(MAY):42-6.
144. Ray U, Sen D, Kar S. Septicaemicmelioidosis. The Journal of the Association of Physicians of India. 2009;57(8):598-9.
145. Riecke K, Wagner S, Eller J, Lode H, Schaberg T. [Pulmonary melioidosis in a German Southeast Asia tourist]. Pneumologie. 1997;51(5):499-502.
146. Saikumar CH, Prabhu MM, Stanley W. A rare case of melioidosis presenting as isolated liver abscess. Journal of the Association of Physicians of India. 2016;64(1):153.
147. Sankar S, Vadivel K, Nandagopal B, Jesudason MV, Sridharan G. A multiplex nested PCR for the simultaneous detection of Salmonella typhi, Mycobacterium tuberculosis, and Burkholderiapseudomallei in patients with pyrexia of unknown origin (PUO) in Vellore, South India. Molecular diagnosis & therapy. 2014;18(3):315-21.
148. Sanklecha MU, Raghavan K, Mehta MN. Melioidosis--rare or overlooked? Indian journal of pediatrics. 1997;64(2):253-5.
149. Saravu K, Kadavigere R, Shastry AB, Pai R, Mukhopadhyay C. Neurologic melioidosis presented as encephalomyelitis and subdural collection in two male labourers in India. Journal of infection in developing countries. 2015;9(11):1289-93.
150. Saravu K, Mukhopadhyay C, Eshwara VK, Shastry BA, Ramamoorthy K, Krishna S, et al. Melioidosis presenting with mediastinal lymphadenopathy masquerading as malignancy: a case report. Journal of medical case reports. 2012;6:28.
151. Saravu K, Mukhopadhyay C, Vishwanath S, Valsalan R, Docherla M, Vandana KE, et al. Melioidosis in southern India: epidemiological and clinical profile. The Southeast Asian journal of tropical medicine and public health. 2010;41(2):401-9.
152. Saravu K, Vishwanath S, Kumar RS, Barkur AS, Varghese GK, Mukhyopadhyay C, et al. Melioidosis--a case series from south India. Transactions of the Royal Society of Tropical Medicine and Hygiene. 2008;102 Suppl 1(SUPPL. 1):S18-20.
153. Satayraddi A, Cherian KE, Kapoor N, Rupali P, Paul TV. Multiple visceral abscesses in a patient with diabetes mellitus: a rare yet corrigible infection: melioidosis. Tropical doctor. 2018;48(1):54-6.
154. Sathiavageesan S. Septicemic melioidosis in a transplant recipient causing graft dysfunction. Indian J Nephrol. 2016;26(5):379-82.

155. Shaaban H, Hallit R, Slim J, Sree A, Sensakovic JW. Reactivation of latent melioidosis presenting with acute pyelonephritis and bacteremia. Avicenna journal of medicine. 2014;4(1):20-1.
156. Shankara BV, Baburaj P, Jacob SS. Study of incidence of Melioidosis for a period of one year and eight months in a Tertiary care Hospital, Kerala, South India. Kerala Medical Journal; Vol 9, No 2 2016.
157. Shaw T, AuCoin D, Dillon M, Chaitana TAK, Patra S, Vandana K, et al. Lateral Flow Assay to diagnose melioidosis in resource constraint setting: a test of choice. 1st South Asian Melioidosis Congress; Manipal, India 2015.
158. Shaw T, Tellapragada C, Eshwara VK, Bhat HV, Mukhopadhyay C. The antibiotics of choice for the treatment of melioidosis in Indian set up. Indian journal of medical microbiology. 2016;34(3):353-4.
159. Shaw T, Tellapragada C, Ke V, AuCoin DP, Mukhopadhyay C. Performance evaluation of Active Melioidosis Detect-Lateral Flow Assay (AMD-LFA) for diagnosis of melioidosis in endemic settings with limited resources. PloS one. 2018;13(3):e0194595.
160. Shenoy V, Kamath MP, Hegde MC, D'Souza T, Mammen SS. Melioidosis and tuberculosis: dual pathogens in a neck abscess. The Journal of laryngology and otology. 2009;123(11):1285-7.
161. Shetty AK, Boloor R, Sharma V, Bhat GH. Melioidosis and pulmonary tuberculosis co-infection in a diabetic. Annals of thoracic medicine. 2010;5(2):113-5.
162. Shetty HS, Mallela AR, Shastry BA, Acharya V. Parietal bone osteomyelitis in melioidosis. BMJ case reports. 2015.
163. Shivbalan S, Reddy N, Tiru V, Thomas K. Systemic melioidosis presenting as suppurative parotitis. Indian pediatrics. 2010;47(9):799-801.
164. Singh A, Grover N, Gupta S, Bhatt P, Sahni A. Disseminated melioidosis. Reviews in Medical Microbiology. 2015;26(3):116-8.
165. Soman R, Davda K, Sunavala A, Chhatwani C, Doshi A. Case Reports: experiences with empiricism in melioidosis. Sri Lankan Journal of Infectious Diseases. 2018;7(Supplement):S42:53.
166. Sood S, Khedar RS, Joad SH, Gupta R. Septicaemic melioidosis: case report from a non-endemic area. Journal of clinical and diagnostic research : JCDR. 2014;8(12):DD01-2.
167. Steinmetz I, Stosiek P, Hergenrother D, Bar W. Melioidosis causing a mycotic aneurysm. Lancet. 1996;347(9014):1564-5.
168. Subaalaxmi MV. Septicemic melioidosis. The Journal of the Association of Physicians of India. 2010;58(2):124; author reply
169. Subbalaxmi MV, Chandra N, Rao MN, Vemu L, Raju YS. *Burkholderia pseudomallei*: an uncommon cause of bacteraemic pneumonia in a diabetic. The Indian journal of chest diseases & allied sciences. 2011;53(3):185-7.
170. Subramanyam P, Palaniswamy SS. Multifocal bone and visceral melioidosis in a cirrhotic patient identified by (99m)Tc MDP bone scan. The American journal of tropical medicine and hygiene. 2014;90(2):191.
171. SugiSubramaniam RV, Karthikeyan VS, Sistla SC, Ali SM, Sistla S, Ram D, et al. Melioidosis presenting as pseudomyxomaperitonei: yet another pretense of the great mimicker: an unreported entity. Surg Infect (Larchmt). 2013;14(4):415-7.

172. SugiSubramaniam RV, Karthikeyan VS, Sistla SC, Ali SM, Sistla S, Vijayaraghavan N, et al. Intra-abdominal melioidosis masquerading as a tubercular abdomen: report of a rare case and literature review. Surg Infect (Larchmt). 2013;14(3):319-21.
173. Swetha RG, Ramaiah S, Sekar K, Anbarasu A. Melioidosis Database (MDB): A comprehensive web-based resource on Melioidosis and gene protein repository for *Burkholderia pseudomallei*. Res J Biotechnol. 2017;12(1):48-55.
174. Tellapragada C, Kamthan A, Shaw T, Ke V, Kumar S, Bhat V, et al. Unravelling the Molecular Epidemiology and Genetic Diversity among Burkholderiapseudomallei Isolates from South India Using Multi-Locus Sequence Typing. PloS one. 2016;11(12):e0168331.
175. Tellapragada C, Shaw T, D'Souza A, Eshwara VK, Mukhopadhyay C. Improved detection of Burkholderiapseudomallei from non-blood clinical specimens using enrichment culture and PCR: narrowing diagnostic gap in resource-constrained settings. Tropical medicine & international health : TM & IH. 2017:n/a-n/a.
176. Thomas J, Jayachandran NV, ShenoyChandrasekhara PK, Lakshmi V, Narsimulu G. Melioidosis--an unusual cause of septic arthritis. Clinical rheumatology. 2008;27 Suppl 2(SUPPL. 2):S59-61.
177. Thurnheer U, Novak A, Michel M, Ruchti C, Jutzi H, Weiss M. [Septic melioidosis following a visit to India]. SchweizerischemedizinischeWochenschrift. 1988;118(15):558-64.
178. Totagi AB, Paramasivan P. Melioidosis: an unusual cause of isolated liver abscess. Trop Gastroenterol. 2014;35(4):261-3.
179. Trivedi P, Tuteja U, Khushiramani R, Reena J, Batra HV. Development of a diagnostic system for *Burkholderia pseudomallei* infections. World journal of microbiology & biotechnology. 2012;28(7):2465-71.
180. Twigg G. Plague in India. Lancet. 1995;345(8944):258.
181. Tyagi P, Shah V, Sharma P, Bansal N, Singla V, Kumar A, et al. Melioidosis Presenting as Fever and Jaundice: A Rare Presentation. J ClinExpHepatol. 2014;4(2):172-4.
182. Udayan U, Dias M. Evaluation of BACTEC blood culture system for culture of normally sterile body fluids. Indian journal of critical care medicine : peer-reviewed, official publication of Indian Society of Critical Care Medicine. 2014;18(12):829-30.
183. Vaid T, Rao K, Hande HM. An intriguing case of locked jaw secondary to melioidosis. BMJ case reports. 2015.
184. Valade E, Thibault FM, Biot FV, Vidal DR. *Burkholderia pseudomallei*: A bacterium which deserves to be better know. Rev Fr Lab. 2009;39(415):49-55.
185. Valsalan R, Seshadri S, Pandit VR. Melioidosis masquerading as enteric fever. Transactions of the Royal Society of Tropical Medicine and Hygiene. 2008;102(SUPPL. 1).
186. Valsalan R, Shubha S, Mukhopadhyay C, Saravu K, Maneesh M, Shastry BA, et al. False-positive widal in melioidosis. Indian journal of medical sciences. 2009;63(10):464-7.
187. Vandana KE, Mukhopadhyay C, Tellapragada C, Kamath A, Tipre M, Bhat V, et al. Seroprevalence of *Burkholderia pseudomallei* among adults in coastal areas in southwestern India. PLoS neglected tropical diseases. 2016;10(4):e0004610.

188. Varughese S, Mohapatra A, Sahni R, Balaji V, Tamilarasi V. Renal allograft recipient with melioidosis of the urinary tract. Transplant infectious disease : an official journal of the Transplantation Society. 2011;13(1):95-6.
189. Vasantha LP, Leela KS. Melioidosis in Andrha Pradesh, India. International Journal of Scientific Research. October 2014 Oct;3(10):453-4
190. Vidyalakshmi K, Chakrapani M, Shrikala B, Damodar S, Lipika S, Vishal S. Tuberculosis mimicked by melioidosis. The international journal of tuberculosis and lung disease : the official journal of the International Union against Tuberculosis and Lung Disease. 2008;12(10):1209-15.
191. Vidyalakshmi K, Lipika S, Vishal S, Damodar S, Chakrapani M. Emerging clinico-epidemiological trends in melioidosis: analysis of 95 cases from western coastal India. International journal of infectious diseases : IJID : official publication of the International Society for Infectious Diseases. 2012;16(7):e491-7.
192. Vidyalakshmi K, Shrikala B, Bharathi B, Suchitra U. Melioidosis: an under-diagnosed entity in western coastal India: a clinico-microbiological analysis. Indian journal of medical microbiology. 2007;25(3):245-8.
193. Vijaykumar GS, Thilakavathy P, Jeremiah SS, Vithiya G. Osteomyelitis of humerus and intramuscular abscess due to melioidosis. Kathmandu Univ Med J. 2016;14(54):184-5.
194. Vishnu Prasad NR, Balasubramaniam G, Karthikeyan VS, Ramesh CK, Srinivasan K. Melioidosis of chest wall masquerading as a tubercular cold abscess. Journal of surgical technique and case report. 2012;4(2):115-7.
195. Viswanathan S, Remalayam B, Muthu V, Kumar S. Diabetes with multiple abscesses disseminated in time and place. Asian Pac. 2012;2(2 SUPPL.):S1209-S11.
196. Viswaroop BS, Balaji V, Mathai E, Kekre NS. Melioidosis presenting as genitourinary infection in two men with diabetes. Journal of postgraduate medicine. 2007;53(2):108-10.
